# Synergistic Effects of pH and Temperature on Dengue Virus Envelope Dimers: Insights from Microsecond Molecular Dynamics Simulations

**DOI:** 10.1021/acs.jcim.6c01334

**Published:** 2026-09-04

**Authors:** Georcki Ropón-Palacios, Luís G. F. Crespi, Ingrid B. S. Martins, Walter Rocchia, Alexandre S. de Araujo

**Affiliations:** † Department of Physics, Institute of Biosciences, Humanities and Exact Sciences, São Paulo State University (UNESP), São José do Rio Preto, São Paulo 15054-000, Brazil; ‡ CONCEPT Lab, 121451 Istituto Italiano di Tecnologia (IIT), Via Enrico Melen, 83, 16152 Genova, Italy

## Abstract

Dengue pathogenesis depends on a conformational rearrangement of the envelope glycoprotein (pE) induced by low pH. However, how pH and temperature cooperate to destabilize the dimeric form of the pE, a fundamental step that enables the formation of the fusion-active trimer, remains unclear. Here, we investigate this synergy for Dengue serotypes of greatest medical relevance, DENV-2 and DENV-3. All–atom molecular dynamics simulations were performed on the microsecond scale, at 28 °C, 37 °C, and 40 °C under pH 5, 6, and 7 using CHARMM36m force fields and complemented by extended coarse-grained molecular dynamics simulations for the DENV-2 system at 37 °C and pH 5, and for the DENV-3 system at 28 °C and pH 5, and at 37 °C and pH 7. Our simulations suggest that increasing the temperature from 28 to 37 °C tends to expand the conformational space at neutral pH, potentially weakening dimer integrity through modulation of the interfacial interaction network. Acidification appears to amplify this effect in a serotype–specific manner: in our simulations DENV-2 required pH 5, while DENV-3 responded at pH 6. Additionally, distance and principal component analyses revealed an asymmetric dissociation pathway, termed the “compensatory embrace”, observed in both all-atom and coarse-grained models. Structural analyses further highlighted the differences in histidine distribution between the serotypes. Moreover, interaction mapping at 37 °C identified candidate interfacial contacts that may act as pH-sensitive latches, maintaining stability at pH 7 but breaking upon acidification. Collectively, these results suggest a potential hierarchy of physicochemical triggers that may contribute to the conversion of dimer into fusion-competent monomers. These interfacial hotspots and the “compensatory embrace” constitute structurally informed targets that, pending validation under native virion conditions, may guide the rational design of antivirals and immunogens, underscoring the value of long-time scale molecular dynamics simulations as a hypothesis-generating tool in drug discovery.

## Graphical abstract

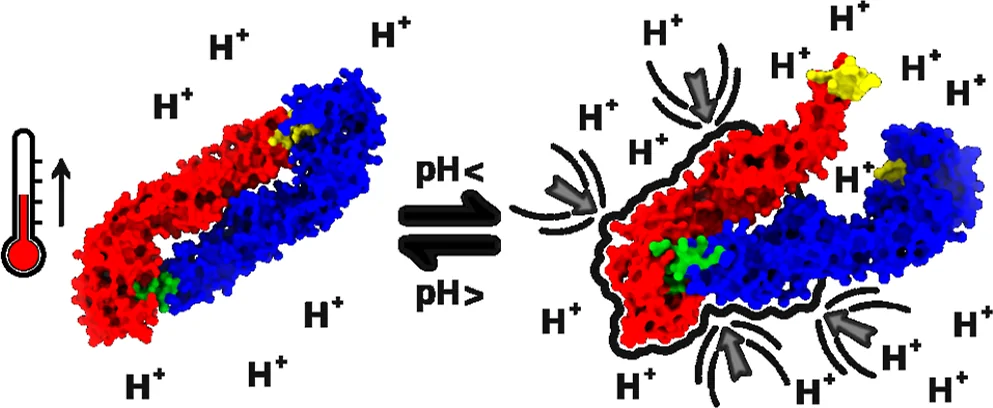

## 1 Introduction

The dengue virus (DENV) has established itself as the most prevalent circulating arbovirus worldwide; in 2024, the disease surpassed 14 million cases and 9500 global deaths.^1,2^ Disease outcomes range from a self-limited febrile illness to dengue hemorrhagic fever (DHF) and dengue shock syndrome (DSS).^3,4^ A major driver of severe manifestations is antibody-dependent enhancement (ADE), whereby subneutralizing antibodies from prior exposure or vaccination facilitate Fc-mediated uptake of a heterologous serotype.^5–7^ The DENV envelope glycoprotein (pE) orchestrates both cell receptor binding and the low-pH membrane–fusion reaction that delivers the viral genome into host cells.^8,9^

Structurally, two envelope glycoproteins (pE) assemble into an antiparallel homodimer that lies flat on the mature virion surface, positioning the ectodomain of the E glycoprotein (ecpE) at the virion surface.^10–12^ Upon endosomal acidification, the dimer is destabilized, exposing a hydrophobic fusion loop in Domain II that inserts itself into the target membrane.^9,10,13–16^ Recent cryo-EM snapshots have captured pH-triggered intermediates, underscoring the ectodomain’s (ecpE) conformational plasticity.^11,12^ Temperature is an equally critical variable.^17^ Conditions corresponding to the mosquito vector (28 °C), normal human physiology (37 °C), and fever (40 °C) modulate virion “breathing”, alter epitope exposure, and weaken pE dimer stability in a serotype-dependent manner. This effect includes temperature-driven structural transitions captured by cryo-EM and HDX-MS, and a strong shift toward monomeric pE at 37 °C that diminishes quaternary-epitope display.^17–20^

Vaccine development is challenged by the existence of four antigenically distinct serotypes (DENV-1-4) and by the requirement to elicit balanced immunity against all of them.^21–23^ The quaternary pE dimer epitope (EDE)—which bridges both protomers—has emerged as a prime target for broad neutralization.^24,25^ However, most structural and computational analyses have focused on single serotypes or acidification in isolation, leaving the combined effects of pH and temperature on pE dimers unexplored.

Here, we attempt to address this challenge using microsecond all–atom and coarse-grained molecular dynamics (MD) simulations, principal component analysis (PCA), and free-energy landscape (FEL) mapping to investigate the ecpE dimer stability of the epidemiologically dominant serotypes DENV-2 and DENV-3 across pH 5, 6, and 7 and at 28 °C, 37 °C, 40 °C. Our results reveal that (i) neutral conditions perturb the structural stability and flexibility, and expand the conformational space in DENV-2 and DENV-3 when the temperature is switched from 28 to 37 and 40 °C (ii) a “compensatory embrace” mechanism that transiently preserves the number of inter-subunit contacts during dissociation, a key process to be targeted, (iii) a temperature- and pH-dependent weakening of the interfacial interaction network, and (iv) serotype-specific histidine networks that could fine-tune the activation threshold. These insights provide a physicochemical roadmap for designing antivirals and immunogens. Specifically, the interactions that are weakened during the transition to an acidic environment emerge as prime targets to be stabilized, thereby preserving protective dimer states and disrupting pathogenic transitions.

## 2 Materials and Methods

### 2.1 System Preparation

The atomic coordinates of dengue virus ecpE dimers (serotypes DENV-2 and DENV-3; PDB IDs 1OAN and 7A3S, respectively) were retrieved from the Protein Data Bank.^26,27^ Histidine protonation states at pH 5, 6, and 7 were assigned using PROPKA3 ^28^ through the PDBReader module of CHARMM-GUI,^29,30^ which also reconstructed missing loops and unresolved atoms. All-atom topologies (PSF) and coordinates (PDB) were generated with the CHARMM36m force field including CMAP corrections.^31,32^ Each ecpE dimer was solvated in a TIP3P water box extending 15 Å beyond the solvent-accessible surface to prevent self-interaction. To reproduce physiological conditions, Na^+^ and Cl^–^ ions were added to neutralize the systems and achieve an ionic strength of 150 mM using the MSTBx v0.5 toolkit (available at: https://github.com/SSiLiq/MSTBx). The final systems comprised approximately 600 thousand atoms for both DENV-2 and DENV-3, including explicit water molecules and counterions, with box dimensions of 184 × 184 × 184 Å and 182 × 182 × 182 Å, respectively.

### 2.2 All-Atom Molecular Dynamics Simulations

All simulations were performed with NAMD 2.14/3.0b6 ^33,34^ following a consistent equilibration and production protocol. Each system was first subjected to 2000 steps of energy minimization using the conjugate gradient algorithm, with positional restraints of 5 kcal mol^–1^ Å^–2^ applied to backbone atoms to relieve steric clashes. Equilibration was carried out in two NPT stages: (1) 2 ns of gradual heating from 60 K to the target temperature (301, 310, or 313 K; approximately 28, 37, and 40 °C) in 1 K increments, while maintaining pressure at 1.01325 bar with a Langevin thermostat and a Nosé–Hoover Langevin piston barostat; (2) 3 ns of NPT at the final temperature and 1.01325 bar under the same restraints and thermostat–barostat settings. Production dynamics were then conducted for 1 μs per system in the *NPT* ensemble. During equilibration and production, a 2 fs integration time step was employed. Long-range electrostatics were evaluated every two steps (fullElectFrequency = 2), and nonbonded interactions every step (nonbondedFreq = 1). All covalent bonds involving hydrogens were constrained (rigidBonds = all). Long-range electrostatics were computed using Particle Mesh Ewald with a 1 Å grid spacing.^35,36^ Short-range van der Waals interactions were truncated at 12 Å with force switching enabled and a switching onset at 10 Å. The Langevin damping coefficient was set to 1 ps^–1^, and piston oscillation and decay periods were set to 200 and 100 fs, respectively. Trajectories were saved every 50 ps (20,000 frames per μs) for all conditions: DENV-2/DENV-3, pH 5/6/7, and temperatures of 301 K (28 °C), 310 K (37 °C), and 313 K (40 °C).

### 2.3 Coarse-Grained Molecular Dynamics Simulations

To evaluate the “compensatory embrace”, previously prepared all-atom structures from the conditions D3T37pH7, D3T28pH5, and D2T37pH5 were selected, as these ones exhibited this mechanism in all-atom simulations, and subsequently mapped to a coarse-grained representation. All-atoms structures were mapped to the Martini force field version 3.0.0.1 using the martinize2 utility.^37^ The secondary structure elements were assigned via DSSP version 3.1.4,^38,39^ and an elastic network model was applied to the backbone beads using a force constant of 700 kJ mol^–1^ nm^–2^ with lower and upper distance cutoffs of 0.0 and 0.9 nm, respectively. Finally, all three systems (D3T37pH7, D3T28pH5, and D2T37pH5) were solvated in cubic boxes of 190 × 190 × 190 Å, strictly preserving the ionic strength, and protonation states of their corresponding all-atom systems.

Coarse-grained molecular dynamics simulations were performed using GROMACS 2024.3 ^40^ patched with PLUMED version 2.9.3.^41,42^ Initially, energy minimization was performed using the steepest descent algorithm until convergence or a maximum of 50,000 steps was reached, with maximum force tolerances of 100 and 50 kJ mol^–1^ nm^–1^ in two consecutive steps, respectively. The systems were equilibrated in the *NPT* ensemble for 50 ns with a 20 fs time step. Position restraints were applied to the protein during minimization and equilibration, with force constant of 2092 kJ mol^–1^ nm^–2^, and a Verlet buffer tolerance of 0.005 was maintained throughout all simulation stages. During equilibration, the system temperature was maintained at each target values using the velocity-rescaling (v-rescale) thermostat^43^ with a coupling time constant of 1.0 ps, applied separately to the protein and nonprotein groups while isotropic pressure was kept at 1.01325 bar using the stochastic cell rescaling (c-rescale) barostat^44^ with a coupling time of 4.0 ps and an isothermal compressibility of 3 × 10^–4^ bar^–1^. Production simulations were performed for 30 μs per replica, with five independent replicas per system, yielding a total accumulated sampling time of 150 μs per system, using a 20 fs time step, with coordinates saved every 500 ps. Temperature was maintained using the v-rescale thermostat (τ = 1.0 ps). Pressure was maintained isotropically at 1.01325 bar using the Parrinello–Rahman barostat^45^ (τ = 12.0 ps; compressibility = 3 × 10^–4^ bar^–1^). Nonbonded interactions were computed using the Verlet cutoff scheme, with neighbor lists updated every 20 steps. van der Waals interactions were truncated at 1.1 nm using the potential-shift-Verlet modifier, while electrostatic interactions were treated using the reaction-field method with a cutoff of 1.1 nm and a relative dielectric constant of 15.

### 2.4 Trajectory Analysis

Trajectory processing was performed with VMD 1.9.4a57 and MDAnalysis 2.7,^46–48^ supplemented with in–house Python, Tcl, and Bash scripts (available at: https://github.com/SSiLiq/Georcki-CompBiology-Biophysics). Structural stability was assessed by calculating the root-mean-square deviation (RMSD) of *C*_α_ atoms relative to the initial configuration. Interface RMSD was computed using only *C*_α_ atoms within 8 Å of the opposite monomer, and per-residue flexibility was quantified using the root-mean-square fluctuation (RMSF). Dimer dissociation events were defined as frames in which the center-of-mass distance between the monomers’ *C*_α_ atoms (in all-atom models) or *BB beads* (in coarse-grained models) exceeded 60 Å. To characterize the “compensatory embrace”, we measured the local distance between the fusion loop (FL) and its cradle alongside the FL solvent-accessible surface area (SASA). These metrics were used to quantify the asymmetric rearrangements driving this mechanism. Conformational dynamics were further explored by principal component analysis (PCA). *C*_α_ trajectories were aligned to the reference structure, and the covariance matrix was constructed to extract the first two principal components (PC1 and PC2), which together captured the largest fraction of atomic variance. Free energy landscapes (FELs) were then computed as

1 $$\Delta G=-k_{B}T\;\ln\left(\frac{P\left(PC1,PC2\right)}{P_{\max}}\right)$$

where *k*_B_ is the Boltzmann constant, *T* the absolute temperature, *P*(PC1, PC2), the joint probability distribution of PC1 and PC2, and *P*_max_ the maximum probability value (i.e., the reference state). For comparison purposes, the FEL minimum of each system was shifted to zero, corresponding to the global lowest free energy state.

Interface contacts were defined as residue pairs with *C*_α_ atoms located within 8 Å across the dimer interface. Contact frequencies were computed for the simulations at 28, 37, and 40 °C at each pH, and only contacts present in all three temperatures for a given pH were retained. GetContacts (available at: https://github.com/getcontacts/getcontacts) was then used to classify interactions into specific types (e.g., hydrogen bonds, π-cation interactions, and salt bridges).

Prior to analyses, coarse-grained trajectories were postprocessed to remove periodic boundary condition (PBC) artifacts using the GROMACS trjconv utility. First, molecules fragmented across periodic boundaries were reconstructed to ensure that both monomers remained intact throughout the simulation (-pbc whole). Trajectories were then unwrapped (-pbc nojump) to ensure continuous diffusive motion during dimer dissociation and avoid PBC artifacts. The Solvent Accessible Surface Area (SASA) of the fusion loop (FL) was calculated for the coarse-grained trajectories using a custom Python script that wraps Tcl routines executed within VMD 1.9.4a57.^46^ The solvent probe radius was set to 1.91 Å, corresponding to the effective size of a small water Martini sphere (sphere T).^49^

The figures were rendered using VMD 1.9.4a57, ChimeraX 1.8, and Matplotlib 3.7.1.^46,50,51^

### 2.5 Statistical Interpretation of the Mobility Metrics

The RMSD, RMSF, PCA/FEL, and contact-frequency analyses reported below should be interpreted as descriptive comparisons among the sampled trajectories rather than as formal tests of statistical significance. In the all-atom simulations, each pH/temperature condition was represented by a single microsecond-scale trajectory, and therefore the time frames are temporally correlated and do not constitute independent replicates. Accordingly, differences in mean RMSD or RMSF that are comparable to the within-trajectory standard deviations are discussed as trends or qualitative shifts in the sampled conformational ensemble. The coarse-grained simulations provide replicate-level support for selected systems, but they were performed only for conditions chosen from the all-atom observations and should not be taken as a complete statistical validation across the entire pH-temperature matrix. Future work using independent all-atom replicas, block-averaged confidence intervals, and/or enhanced-sampling approaches will be required to quantify the statistical significance of these mobility differences.

## 3 Results and Discussion

In this section, we present and discuss our findings on how pH and temperature jointly modulate the structural dynamics of the dengue virus ecpE dimer in serotypes DENV-2 and DENV-3. Our simulations suggest that acidic pH and elevated temperature remodel the conformational landscape of the ecpE, indicating potential alterations in its ability to mediate host cell entry and evade the host immune system, while also suggesting possible targets for the action of small-molecule inhibitors of its fusogenic activity. Elucidating these physicochemical triggers is therefore critical for the rational design of potent neutralizing antibodies, antiviral drugs, and next-generation vaccine candidates.

### 3.1 Structural Comparison of the pE Ectodomain and Histidine Distribution in DENV-2/3

Because histidine residues are the primary titratable amino acids within the considered pH range, they likely play a crucial role in driving pE dimer dissociation during the viral transition from physiological to acidic endosomal environments. Therefore, their specific structural distribution across serotypes is of great interest. In Figure 1A, we show the high-resolution X-ray crystal structures of the ecpE for DENV-2 (PDB ID 1OAN) and DENV-3 (PDB ID 7A3S) side by side in cartoon representation. Domains I (red), II (yellow), III (blue), and the fusion loop (purple) were highlighted, and all histidines were rendered as sticks and labeled by residue number.

**Figure 1:**
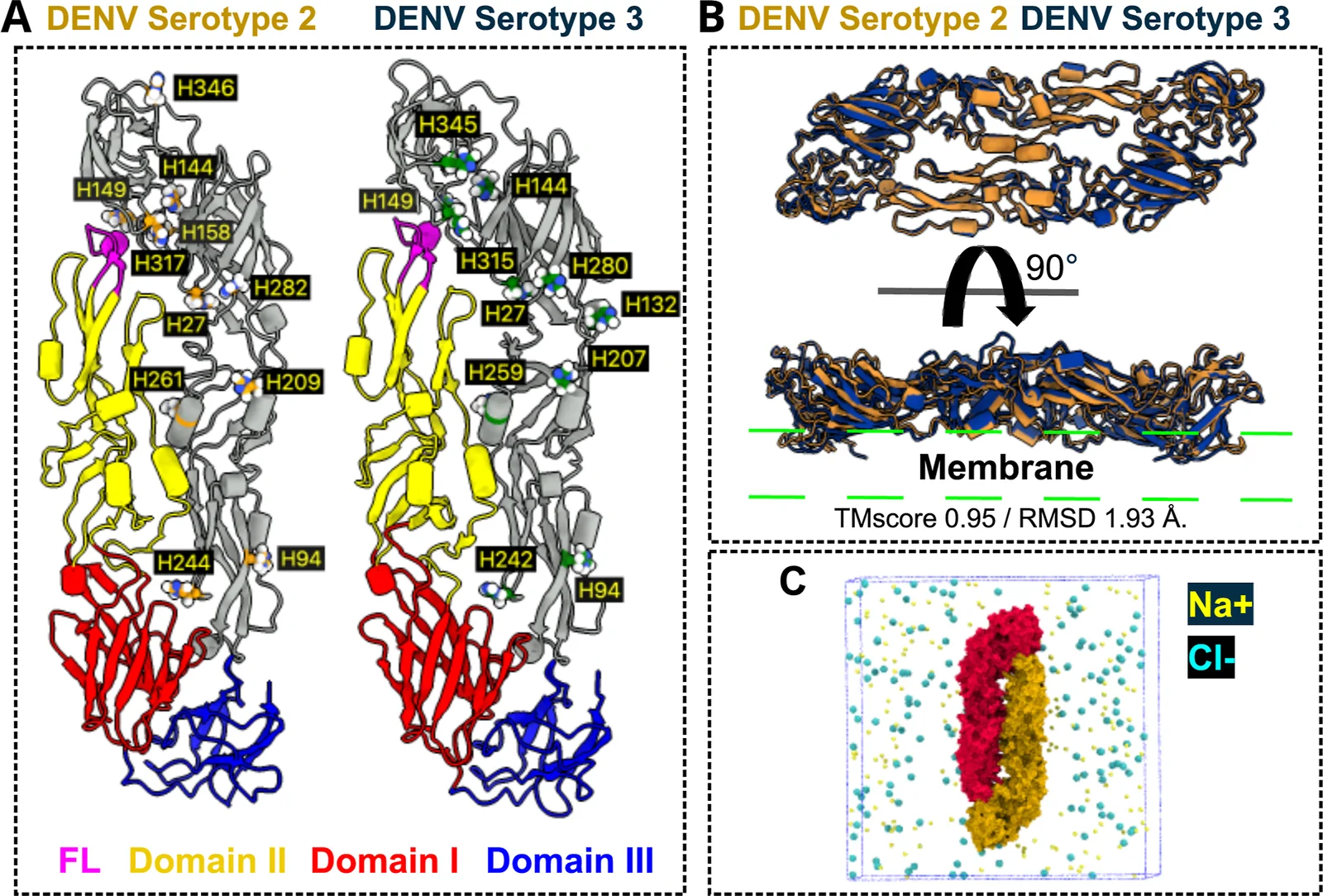
Experimental models of the ecpE dimer from DENV-2 and DENV-3 serotypes. (A) Complete crystallographic structures of the ectodomain, with the DENV-2 and DENV-3 dimers displayed side by side. Domains I (red), II (yellow), and III (blue), as well as the fusion loop (purple), are highlighted in distinct colors. All histidines are shown as sticks and labeled with their corresponding residue numbers. (B) Structural superposition of the ectodomains of DENV-2 and DENV-3. TM-score and RMSD values were obtained with the TM-align program.^52^ (C) Solvated model of pE. Na^+^ and Cl^–^ ions are shown as van der Waals spheres; water molecules were omitted for clarity.

Our analysis reveals subtle differences in the spatial arrangement of histidines along the monomer, in agreement with previous reports.^13^ Both serotypes conserve four histidines adjacent to the fusion loop: His144, His149, His158, and His317 in DENV-2, and His144, His149, His315, and His345 in DENV-3; among them, His144 and His317 (His315 in DENV-3) have been highlighted in the literature as major pH sensors at the DI–DIII interface.^13,14^ Although His149/His158 in DENV-2 and His149/His345 in DENV-3 lack direct experimental validation as essential pH sensors, we hypothesize that these secondary histidines, together with the others, may fine-tune the acid-dependent activation threshold for dimer dissociation. This process likely occurs in a serotype-specific manner without perturbing the global fold. This effect likely arises through a network of interactions around the fusion loop, as suggested in the literature.^53^ For example, His244 in DENV-2 (His242 in DENV-3) has been proposed to mediate interactions with Asp63 of prM and to play a role in viral maturation rather than fusion.^54,55^ Given that these mechanistic interpretations critically depend on the protonation behavior of histidine residues, we acknowledge that static protonation states assigned by PROPKA3—while standard for large-scale MD—may not fully capture the dynamic titration effects (e.g., as in *CpHMD* simulations) occurring during conformational transitions. Consequently, while our results provide a strong structural basis, they should be interpreted with caution, as future *CpHMD* simulations would be necessary to fully reinforce these mechanistic conclusions.

We also superimposed the two ectodomains using TM-align (Figure 1B), yielding a TM-score of 0.95 and an RMSD of 1.93 Å, which confirms strong conservation of the overall fold as reported in structural and bioinformatics studies,^56,57^ despite 25–40% sequence divergence between serotypes.^58,59^ A closer examination of the structures, particularly the surface-exposed loops, revealed that most of the structural variability is localized to Domains I and III, whereas Domain II remains largely unchanged. We propose that these subtle structural differences underlie reported variations in thermal stability, immunogenicity,^56^ receptor affinity, and potentially dimer stability and dynamics.^56,63^ However, further theoretical and experimental studies will be required to rigorously test and validate this hypothesis. Importantly, despite the globally conserved fold, minor sequence divergences pose challenges for the rational design of vaccines, neutralizing antibodies, and small-molecule inhibitors.

Finally, Figure 1C illustrates the solvated crystallographic systems we prepared for our all-atom MD simulations. Each protomer is shown in a distinct surface color; Na^+^ and Cl^–^ ions appear as van der Waals spheres within the dashed simulation box; and water molecules have been omitted for clarity. This structural framework underpins our dynamic analyses, enabling us to correlate specific conformational responses with variations in pH and temperature.

### 3.2 Dimer Stability under pH and Temperature Variations

To assess how pH and temperature jointly influence the stability of the ecpE dimer in DENV-2 and DENV-3, we performed all-atom molecular dynamics simulations at 28 °C, 37 °C and 40 °C across pH 5, 6, and 7. These serotypes were chosen for their epidemiological relevance^64–66^ and for subtle primary-sequence variations that, as confirmed in Figure 1B, do not alter the global ectodomain fold but may drive distinct dynamic behaviors. In Figure 2A, each monomer is rendered in a distinct color and both frontal and posterior views of the dimer are shown.

**Figure 2:**
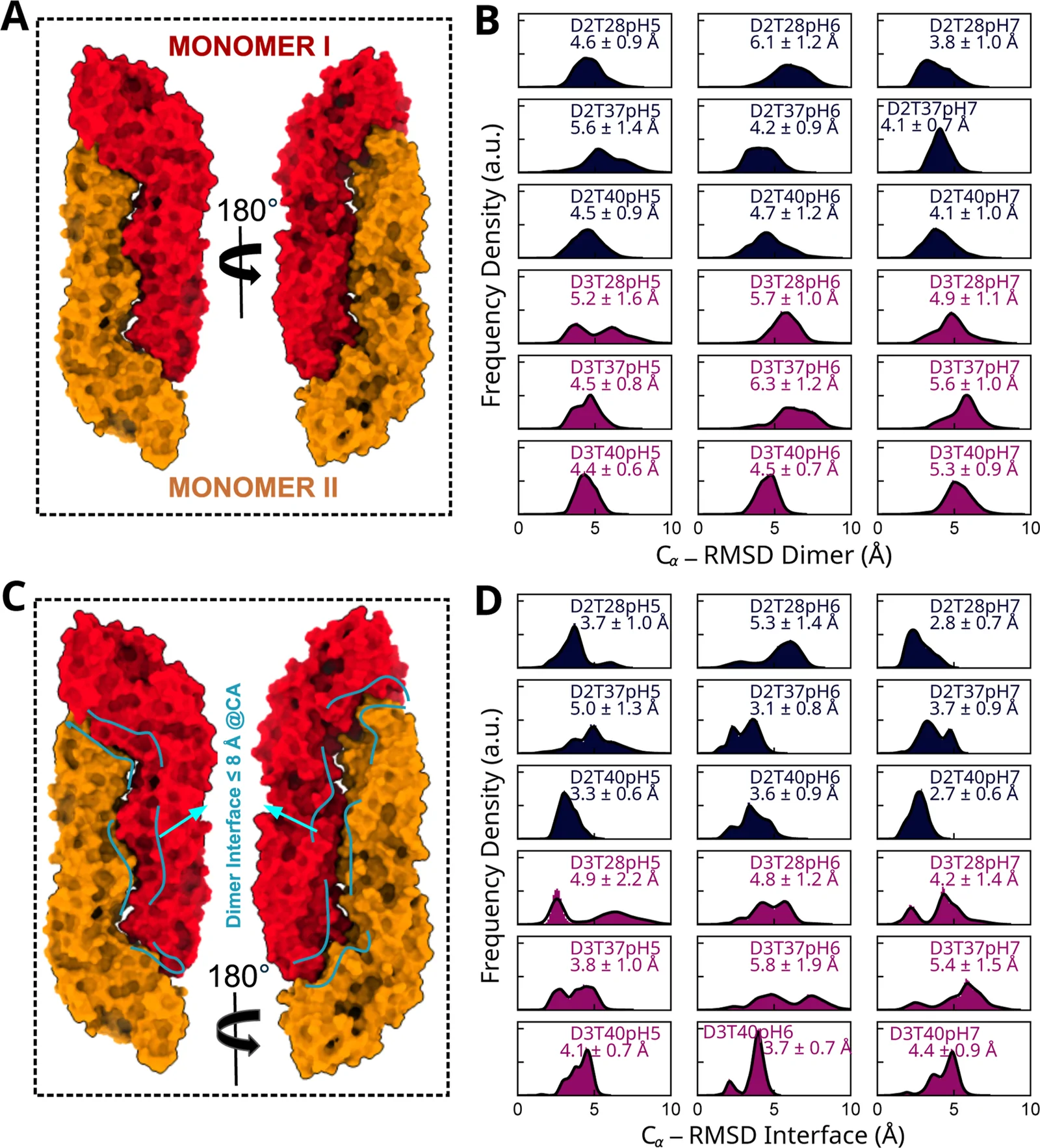
Conformational stability of the ecpE dimer from DENV-2 and DENV-3 serotypes. (A) Graphic representation of the dimer, with each monomer highlighted in a different color and shown in both front and back views. (B) Global *C*_α_ RMSD histograms, calculated from simulations performed at 28 °C, 37 °C, and 40 °C under pH 5, 6, and 7. (C) Interface selection diagram indicating the residues whose *C*_α_ atoms are within ≤8 Å of the other protomer. (D) Interface RMSD histograms (*C*_α_) for the residues considered in panel (C), under the same temperature and pH conditions.

Our global RMSD histograms (Figure 2B) suggest two apparent regimes: (i) stable fluctuations around a well-defined conformational basin, reflected by narrow, unimodal peaks; and (ii) transitional deviations associated with dimer destabilization, characterized by broader and multimodal distributions. It is important to note that the large standard deviations indicate overlapping conformational distributions across different conditions. Therefore, rather than absolute structural divergences, these average RMSD values reflect shifts in the dynamic propensity of the dimer. These patterns are consistent with the combined influence of pH and temperature on dimer stability.

At pH 5, DENV-2 reaches its highest RMSD (5.6 ± 1.4 Å) at 37 °C—an endosomal temperature typical of the human host^55^—consistent with fusion of the NGC DENV-2 strain peaking at pH 6.2 or below.^55,67^ In contrast, DENV-3 exhibits its maximum RMSD (6.3 ± 1.2 Å) at 37 °C only at pH 6, aligned with experimental reports^18^ and suggesting how differences in histidine distribution may modulate each serotype’s pH sensitivity. Interestingly, at pH 6 we observe for DENV-2 peaks at 28 °C RMSD (6.1 ± 1.2 Å), reflecting mosquito-like conditions, whereas DENV-3 remains maximal at 37 °C, in agreement with binding studies reporting optimal attachment between 37 and 40 °C, with 37 °C being most favorable.^68^ At neutral pH, DENV-2 maintains elevated RMSD (4.1 ± 0.7/1.0 Å) at both 37 and 40 °C, while DENV-3′s maximum RMSD (5.6 ± 1.0 Å) was observed only at 37 °C. This behavior concurs with the literature indicating that neutral pH and physiological temperatures favor dimer-to-monomer dissociation; under these conditions, DENV-3 displays a higher tendency for the monomeric form.^18^

### 3.3 Local Interface Perturbation and Residue Flexibility

To gain deeper insight, we next analyzed the dimer interface, defined as pairs of *C*_α_ atoms within 8 Å across monomers (Figure 2C), and extracted interface RMSD histograms (Figure 2D) to capture local contact loosening. For DENV-2 at pH 5 and 37 °C, the mean interface RMSD is 5.0 ± 1.3 Å, pointing to a trend of local contact loosening; at pH 6 and 28 °C, the peak shifts similarly (5.3 ± 1.4 Å), suggesting that early-stage dissociation could occur under mosquito-like conditions.^59^ By contrast, at physiological pH (6–7) and 37–40 °C, interface RMSD remains near 3 Å, but rises to approximately 3.7 Å at pH 7 and 37 °C before settling at 2.7 ± 0.6 Å at 40 °C, suggesting a possible thermal “switch” between vector and host environments.

In DENV-3, the largest observed interface RMSD (5.8 ± 1.9 Å) occurs at pH 6 and 37 °C; at pH 5 and 28 °C it remains elevated (4.9 ± 2.2 Å), shifting to 4.4 ± 0.9 Å at pH 7 and 40 °C—suggesting that neutral and febrile conditions may favor the destabilization of this serotype, although the broad distributions warrant a cautious interpretation. Under physiological pH, raising the temperature from 28 to 37 °C reproduces the same apparent temperature-dependent change pattern, with RMSD of 4.2 ± 1.4 Å at 28 °C and 5.4 ± 1.5 Å at 37 °C. However, cryo-EM data of mature virions show that soluble DENV-3 ectodomains incubated at 37 °C remain structurally similar to those at 28 °C,^69^ suggesting reversible thermal “breathing” response in solution without irreversible unfolding, in contrast to the NGC strain of DENV-2. In light of this apparent paradox, we hypothesize that the thermal “switch” operates differently in soluble dimers versus the virion surface-potentially modulated by viral components such as a protein M, the lipid bilayer and varying by strain–and therefore must be contextualized to the specific structural level under investigation to avoid confusion with the “breathing” in virion context.

In the light of the thermal “switch” described above, Figure 3 presents per-residue RMSF profiles for each monomer at 28 °C, 37 °C and 40 °C across pH 5, 6, and 7. While global RMSF values are statistically comparable between serotypes due to overlapping standard deviations, an analysis of the mean fluctuation profiles reveals distinct tendencies: at pH 7 and 37 °C, DENV-2 shows elevated flexibility in Domains I and II (residues approximately 100–200), which may assist in fusion-related rearrangements, and at 40 °C in Domains I and III (e.g., residues approximately 280–296 and 380–394). Under pH 5 and 37 °C, DENV-2 exhibits its highest RMSF (3.0 ± 1.2 Å) in chain B. In contrast, DENV-3 reaches maximum flexibility (2.8 ± 1.0 Å) in chain B at pH 5 and 28 °C, followed by pH 6 at 37 °C (2.5 ± 1.0 Å) in chain B and pH 7 at 37 °C (2.5 ± 1.0 Å) in chain C, with Domain III tended to exhibit the highest flexibility in several of the sampled conditions. Interestingly, this pattern of specific peaks points toward a potential asymmetric behavior: as one monomer’s flexibility increases, the partner’s decreases, indicative of an asymmetric dissociation mechanism that we explore in the next section.

**Figure 3:**
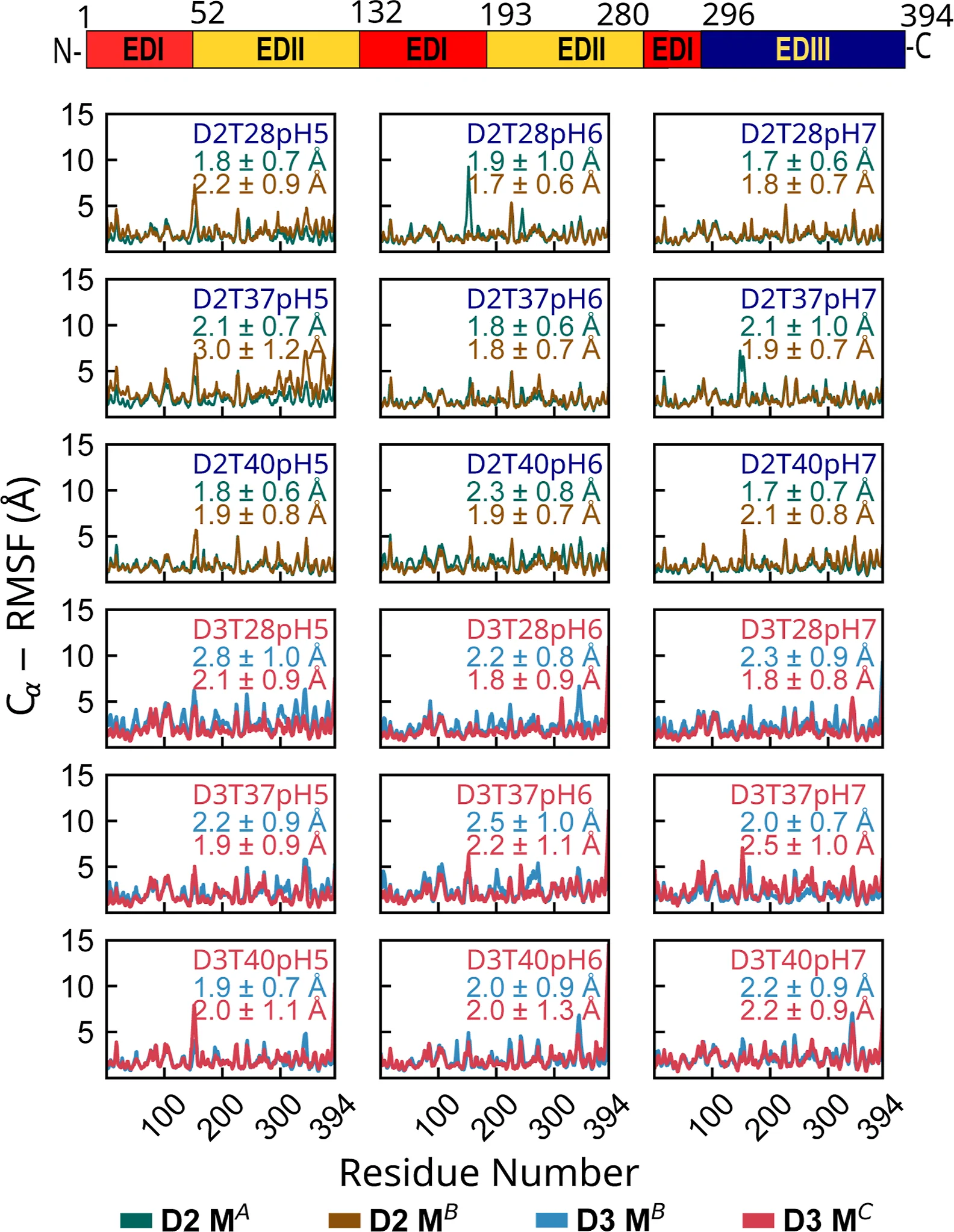
Residual flexibility profile (RMSF) of the E protein from DENV-2 and DENV-3 serotypes. RMSF curves along the sequence for each monomer, obtained from simulations at 28 °C, 37 °C, and 40 °C under pH 5, 6, and 7. Above, a schematic diagram of domains DI–DIII indicates the location of residues to facilitate interpretation. In the legend of the graph, the abbreviations D2M^A^, D2M^B^, D3M^B^ and D3M^C^ indicate the viral serotype (D2: DENV-2; D3: DENV-3) and the corresponding monomer chain, represented by M^X^, where X = A, B, C.

### 3.4 pH and Temperature Trigger ecpE Dimer Separation Events within 1 μs of All-Atom Molecular Dynamics

In order to observe the onset of dissociation, we monitored the distance between the centers of mass of the monomers (*d*_*M*→*M*_) over 1 μs of all-atom molecular dynamics simulations; a duration long enough to observe selected rearrangements in this dimer.^70^ Based on the crystallographic dimer structure, in which the intermonomer distance is approximately 50 Å, we defined 60 Å as the threshold for dimer dissociation (Figure 4A, green line). This threshold incorporates a 20% tolerance margin to account for possible structural fluctuations at the dimer interface during the simulations. Distances beyond this value indicate the onset of separation, as they exceed the native spacing observed in the crystal. In Figure 4A, we present d_*M*→*M*_ histograms for DENV-2 and DENV-3 under each pH–temperature combination; in Figure 4B, we display representative snapshots of the three trajectories that exceeded this threshold.

**Figure 4:**
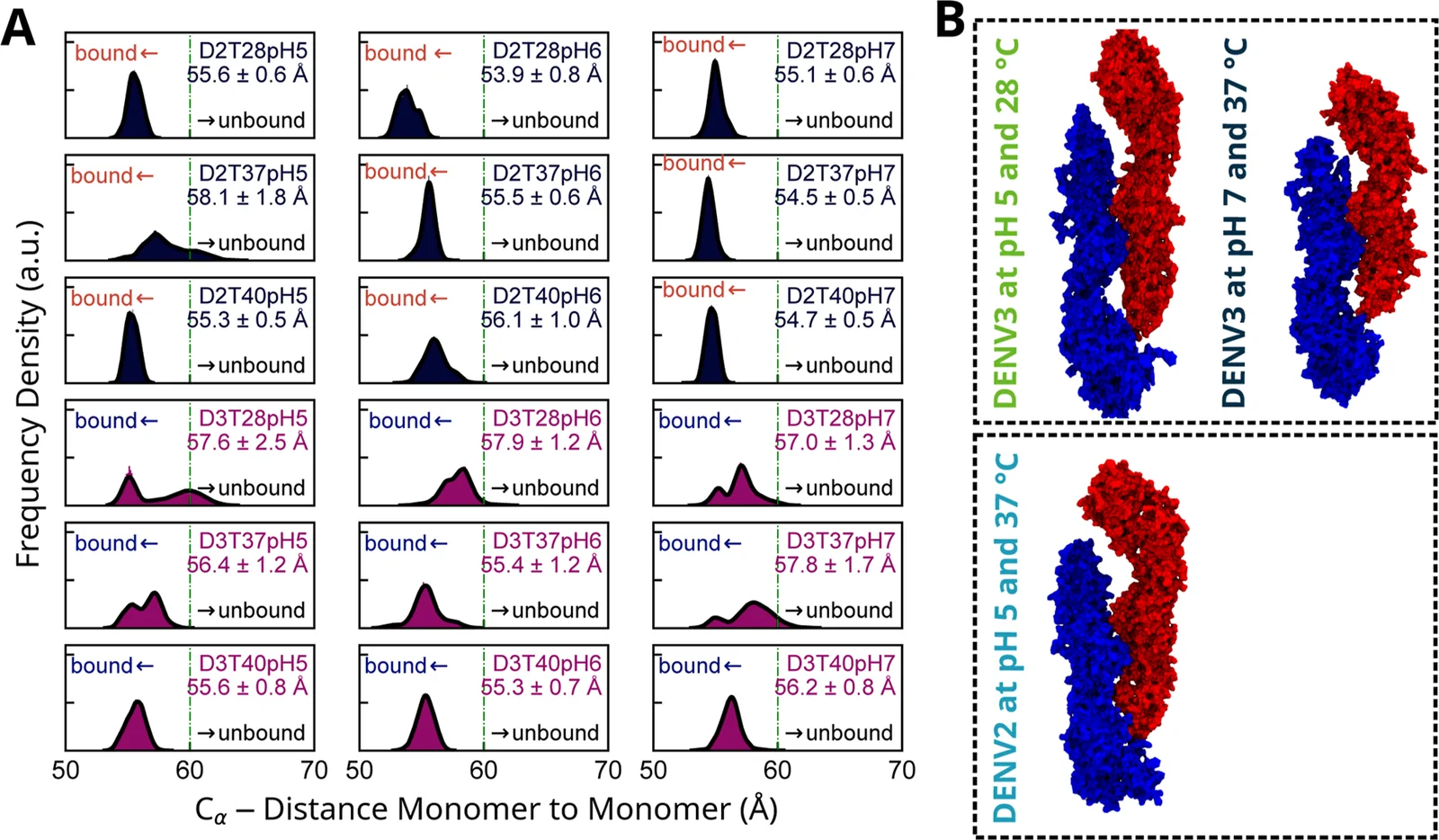
Dissociation dynamics ecpE dimers from DENV-2 and DENV-3 under different pH and temperature combinations. (A) Histograms of the distance between monomer centers of mass (*d*_*M*→*M*_) obtained after 1 μs of molecular dynamics. The green line marks 60 Å, the threshold value defined as the onset of dimer dissociation. (B) Representative structural snapshots of the three trajectories that exhibited dimer dissociation.

Although dimer dissociation is intrinsically stochastic and its precise kinetic or thermodynamic properties cannot be determined from the present sampling, our microsecond simulations captured spontaneous dissociation events in three independent trajectories: DENV-2 at pH 5 and 37 °C, DENV-3 at pH 5 and 28 °C and DENV-3 at pH 7 and 37 °C (Figure 4). These events allow us to describe representative structural features of dissociation.

In this context, our results agree with the evidence that low pH acts as a trigger for E protein fusion, promoting conformational changes in the ectodomain,^13^ while physiological temperatures favor the dimer-to-monomer transition in both DENV and ZIKV.^18,64^ By analyzing histograms and structures extracted from the trajectories; we observed a process consistent with a mechanism of asymmetric dissociation: while one monomer loses contacts, with its Domains I and III moving away from Domain II of the opposite monomer, the other side of the dimer tightens as the adjacent monomer approaches. This rearrangement, which we term a “compensatory embrace”, can preserve or even slightly increase the overall number of inter-subunit contacts, thereby masking partial separation (Figure 4B, see Videos S1–S3 in the Supporting Information).

To assess the recurrence of the “compensatory embrace” mechanism, we performed coarse-grained molecular dynamics simulations using five independent replicas for each of the D2T37pH5, D3T28pH5, and D3T37pH7 systems. While the separation profiles between all-atom and coarse-grained models were generally consistent (Figures 4A and 5A), we observed that the D2T37pH5 system did not exceed the 60 Å dissociation threshold. We attribute this outcome to either the inherent limitations of the coarse-grained model or to limited conformational sampling for this specific system. In contrast, the DENV-3 models (D3T28pH5 and D3T37pH7) exhibited complete dimer dissociation in 2 out of 5 replicas, a result that aligns with the previously reported structural instability of this serotype.^18,71^

**Figure 5:**
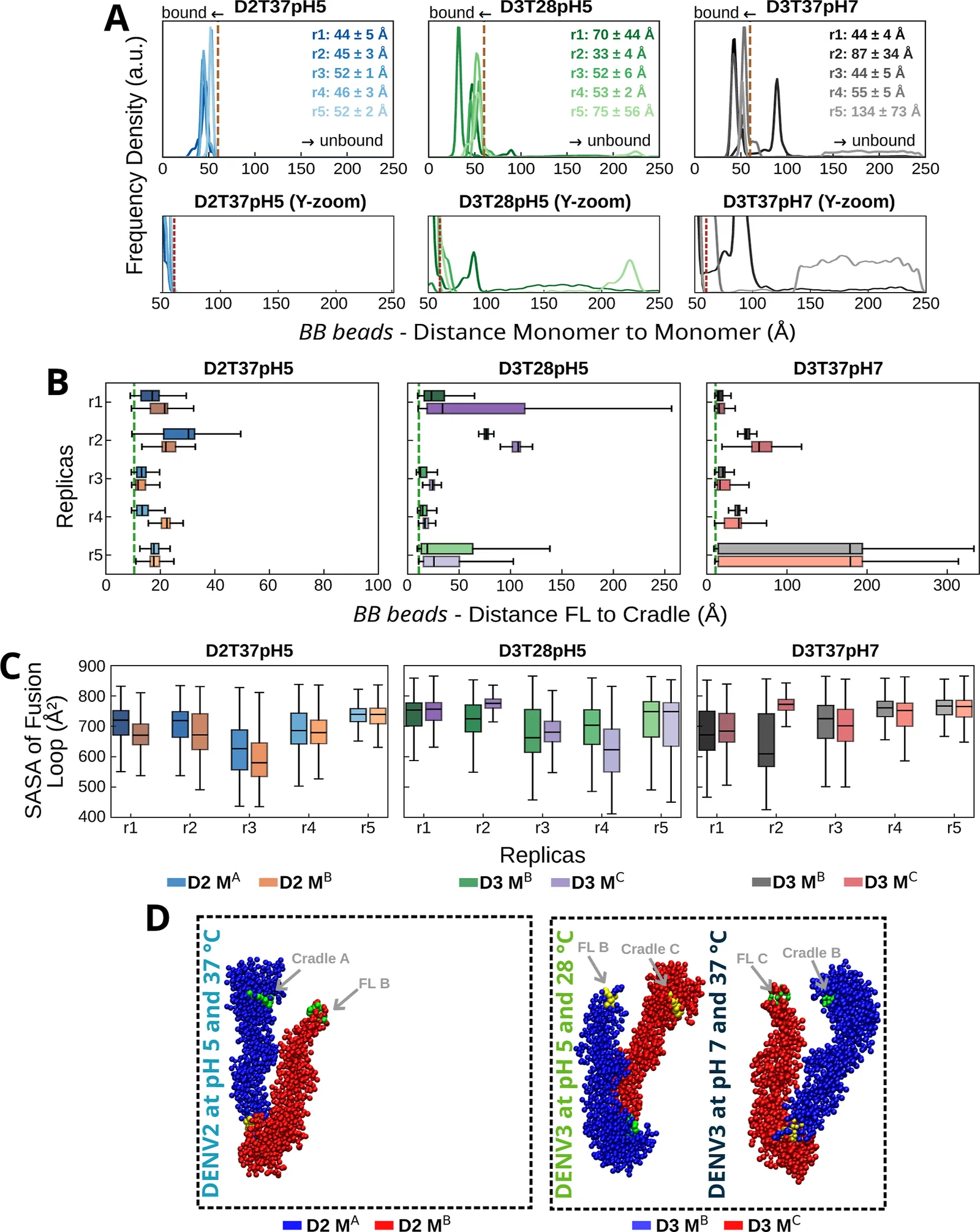
Coarse-grained molecular dynamics for the systems that exhibited the “compensatory embrace” mechanism in all-atom simulations. (A) Distance between the centers of mass of each monomer; the dashed line at 60 Å marks the threshold defined for the start of dimer dissociation. (B) Distance between the centers of mass of the FL (residues 98–110) and its respective “cradle” (residues 310–320) for chains A and B in DENV-2 and for chains B and C in DENV-3; the dashed line represents the reference value of the crystal structure. (C) Solvent-accessible surface area (SASA) of the FL for each replica and for each chain (color code shown below) (D) Observation of the “compensatory embrace”, both captured in the first replicate (r1).

Despite the lack of global separation in the D2T37pH5 model, an analysis of the distance between the fusion loop (FL) (Figure 5B) and its “cradle” revealed asymmetric movement in 4 out of 5 replicas. This asymmetric behavior is one feature of the proposed “compensatory embrace” mechanism and was also observed in the DENV-3 models. Notably, although one D3T28pH5 replica adopted a crossed conformation as an outlier, the overall mechanism remained observed in multiple replicas. Finally, the solvent-accessible surface area (SASA) analysis (Figure 5C) of the FL corroborated the asymmetric profile identified by the distance metrics, further supporting the recurrence of the proposed “compensatory embrace” mechanism, as illustrated in Figure 5D (see also Videos S4–S6 in the Supporting Information).

Importantly, despite the stochastic nature of dimer dissociation, we found that the “compensatory embrace” (Figures 4B and 5) constituted a consistent structural pathway for dimer dissociation (see Video S7 in the Supporting Information). While this mechanism emerges as a plausible target for molecular stabilization, further investigations are required to corroborate whether it persists within the context of the full viral envelope. Future studies must determine if the presence of the M protein and the lipid bilayer modulates these transitions or if the “embrace” primarily arises from the structural degrees of freedom available to soluble ectodomains in solution.

### 3.5 Effect of pH and Temperature on the Essential Dynamics of the ecpE Dimer

To elucidate how pH and temperature jointly modulate the essential motions of the ecpE dimer in DENV-2 and DENV-3, we applied an integrated principal component analysis (PCA) and free energy landscape (FEL) approach (Figure 6A–D). In Figure 6A, each point represents a conformation sampled over 1 μs, colored according to its simulation time. At neutral pH, in the sampled trajectories, increasing the temperature from 28 to 37 °C was associated with an expansion of the conformational space explored: in DENV-2, the originally compact cloud split into two connected clusters, whereas DENV-3 dispersed into three distinct regions. Consistently, the FELs in Figure 6B show that DENV-2 shifts from a single basin of approximately 2.3 *k*_B_ *T* at 28 °C to two minima separated by approximately 0.8 *k*_B_ *T* at 37 °C, while DENV-3 displays three shallow basins with internal barriers below 0.5 *k*_B_ *T*.

**Figure 6:**
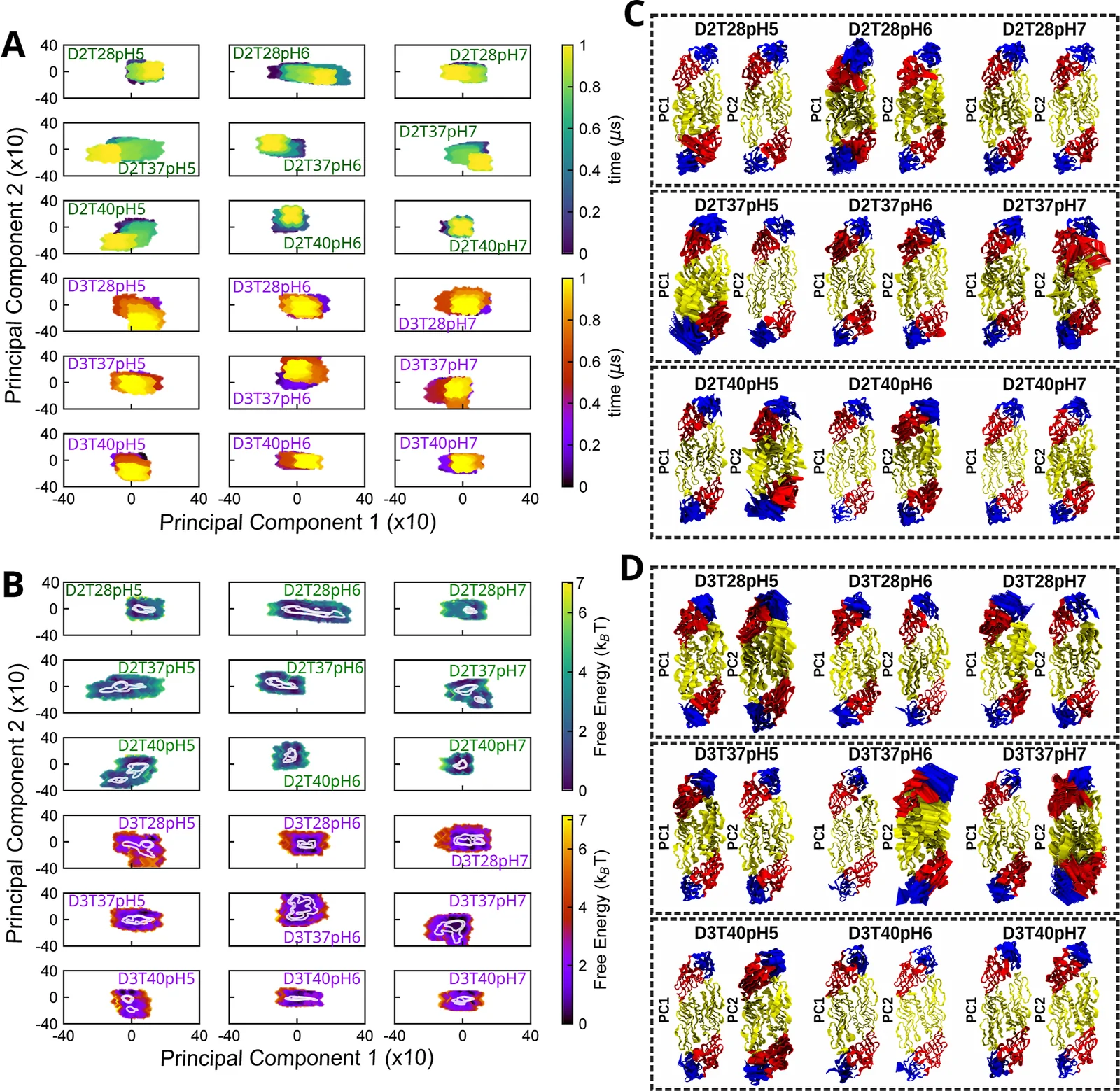
Combined effect of pH and temperature on the essential dynamics of the ecpE dimer from DENV-2 and DENV-3 serotypes. (A) PCA projections; each point represents a conformation sampled over 1 μs, with a color gradient indicating temporal evolution. (B) Free energy landscapes (FEL) on the PC1 × PC2 plane. (C) Dominant modes of DENV-2. (D) Dominant modes of DENV-3. In panels (C,D), domains DI, DII, and DIII are colored according to the conventional E-protein palette defined previously.

We next examined the extreme PCA vectors (Figure 6C,D), which indicate that PC1 is dominated by the rotation of Domain III and PC2 by an asymmetric swing of Domain I around the DI–DIII interface. These motions underpin our “compensatory embrace” hypothesis, in which the retraction of DIII in one monomer is offset by an advance of DI in its partner, thereby preserving inter-subunit contacts even as the dimer approaches monomerization.^64^ In DENV-3, this effect is even more pronounced, consistent with the substantial monomeric fraction observed at 28 °C that further increases at 37 °C.^18,71^ Such large-scale displacements likely hinder recognition of quaternary epitopes and drive researchers to engineer stabilizing mutations, an insight that is valuable for antibody design and subunit vaccine development.^24,71^

Strikingly, at neutral pH, raising the temperature from 28 to 37 °C amplifies motions in Domains I and II along PC2. This effect is more pronounced in DENV-3 and may induce contact loss and favor dimer dissociation during the thermal transition. These findings align with thermodynamics assays reporting that the dimer interface exhibits a high exothermic enthalpy (Δ*H*) dependent on salt bridges.^18,71^ Consequently, the complex may be more susceptible to dissociation upon heating.

At pH 6, we observe a conformational “lock”, which, as noted above, may reflect the absence of additional viral components in the modeled system or limited sampling in our simulations. For DENV-2 at 28 °C, the FEL collapses into a single basin of approximately 1.6 *k*_B_ *T* and the PCA cloud contracts to a small radius, with fluctuations confined to Domain I and minimal motion of DIII (Figure 6C). An identical pattern emerges for DENV-3 at 37 °C, suggesting that mildly acidic conditions reduce large-scale rearrangements.

By contrast, acidification to pH 5 at 37 °C remodels the FEL into interconnected shallow minima (<1.2 *k*_B_ *T*) and projects Domain II toward solvent—a prelude to progressive release of DIII (Figure 6A–D). This opening is more pronounced in DENV-2 than in DENV-3. During this process, DI and DII swing in opposite directions to re–establish the “compensatory embrace”; once these motions reach their maximum amplitude, we propose that the interface loses cohesion and the dimer crosses the 60 Å separation threshold (Figure 4), with acidic pH as the conformational trigger for dimer dissociation.^72^

### 3.6 Effect of Temperature on Molecular Interaction at the DENV E Protein Dimer Interface

To elucidate, in detail, how temperature modulates the interaction network at the DENV-2 and DENV-3 ecpE dimer interface, we mapped all interfacial contacts (Figure 7A,B). In Figure 7A,B, blue highlights indicate residue pairs whose contact frequency drops between the mosquito vector (28 °C) and human physiological (37 °C) temperatures. Curiously, a subset of these interactions shows a partial recovery in frequency at the febrile temperature of 40 °C.

**Figure 7:**
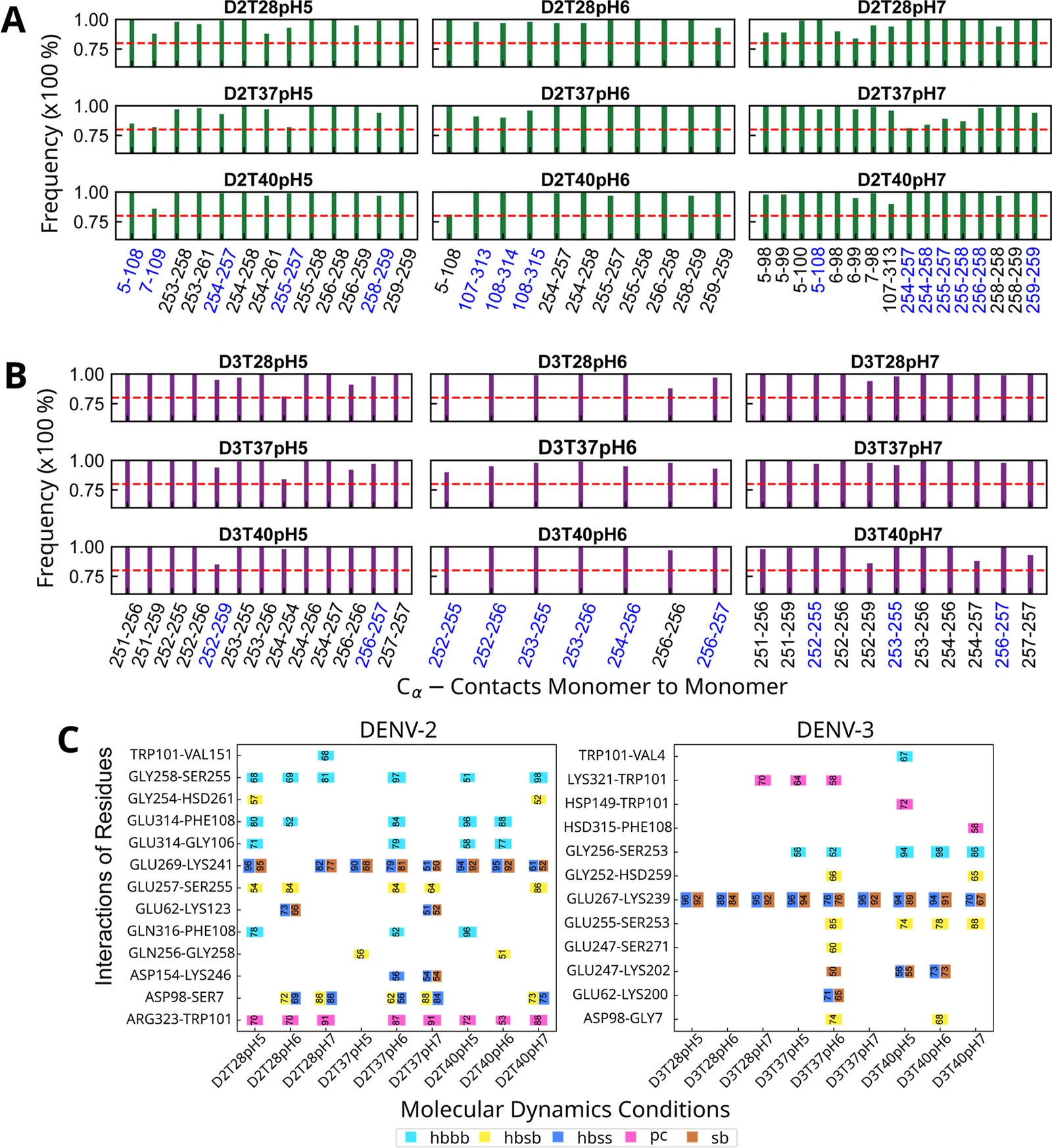
Combined effect of pH and temperature on the molecular interactions of the ecpE dimer from DENV-2 and DENV-3 serotypes. (A,B) Frequency of common contacts across different temperatures at a given pH. Blue-colored pairs indicate residues whose contact frequency is sensitive to temperature changes from 28 °C (vector) to 37 °C (host). (C) Quantification in DENV-2 and DENV-3 of backbone–backbone (hbbb), side chain–backbone (hbsb), and side chain–side chain (hbss) hydrogen bonds, as well as π–cation (pc) and salt bridges (sb), under different simulation conditions.

Next, to characterize the underlying molecular mechanisms in these dengue variants, we evaluated and quantified hydrogen bonds–backbone–backbone (hbbb), side chain–backbone (hbsb), and side chain–side chain (hbss)-as well as π–cation (pc) interactions and salt bridges (sb) (Figure 7C).

Comparing vector conditions (28 °C) with host-like temperatures (37 °C) and febrile (40 °C), we observed temperature-dependent reorganization of interfacial interactions. While several contacts weaken at 37 °C relative to 28 °C, some interactions at 40 °C exhibit partial recovery (Figure 7A,B) or altered occupancy (Figure 7C), indicating a nonmonotonic response under thermal stress. Given the small temperature difference between 37 and 40 °C, these variations should be interpreted cautiously. Nevertheless, both host-like conditions deviate from the vector temperature, supporting the notion that elevated temperature appears to promote structural rearrangement at the dimer interface.

To further explore this, we analyzed the effect of temperature at pH 7 for DENV-2: raising the temperature from 28 to 37 °C reduced the Asp98–Ser7 (DII–DI interface) hbss frequency from 86% to 84%, falling to below 76% at 40 °C for both hbsb and hbss. We also found that the Asp154–Lys246 (DI–DII) salt bridge and hbss showed reduced occupancy, retaining only 54% occupancy at 37 °C and disappearing at 40 °C, consistent with the exothermic nature of salt bridges, whose stability decreases as temperature rises.^18^ In the Glu269-Lys241 (DII–DII) pair, we recorded decreases of over 20 percentage points in hbss and sb from 28 to 37 °C, with the greatest reduction occurring between 28 and 37 °C. This pair lies in the intradimer interface (residues 238–260), which shows temperature-dependent disruption in hydrogen–deuterium exchange assays.^17^ Additionally, the Gly258–Ser255 (DII–DII) hbbb contact was present only at 28 and 40 °C, suggesting a high–temperature conformational rearrangement, while Trp101–Val151 (DII–DI) remained absent at 37 and 40 °C, indicating loss of hbbb under those conditions. In the DENV-3 dimer, Glu267-Lys239 (DII–DII) maintained stable sb and hbss at 37 °C but lost over 20 percentage points of both at 40 °C, and Lys321-Trp101 (DIII–DII; pc) disappeared at both elevated temperatures.

### 3.7 Identification of pH-Sensitive Interfacial Contacts as Targets for Stabilization

To identify candidate targets for therapeutic intervention, such as molecular glues or nanobodies designed to lock the dimer in its prefusion state, we analyzed the specific interaction networks that maintain dimer integrity at physiological temperature (37 °C) under neutral conditions (pH 7) but are compromised upon acidification (pH 6 and/or 5).

In DENV-2, the analysis of contacts revealed three critical stabilizing regions susceptible to acidic pH. The first two regions, located at the DI–DII interface (involving residues 5–7 interacting with the 97–100 fusion loop region) and the central DII–DII interface (centered on residue 258), lose cohesion at both pH 6 and pH 5. Crucially, a third region bridging DII and DIII (involving residues 107 and 313) dissociates exclusively at pH 5. These observations are consistent with a hierarchical disassembly mechanism for DENV-2, where initial loosening occurs at pH 6, but the critical DI–DIII “latch” is only released at pH 5, fully aligning with the maximal structural deviation observed for this serotype under endosomal-like conditions.

In contrast, DENV-3 exhibited a more concentrated and specific sensitivity. The loss of hydrophobic contacts was confined to a single cluster within the central DII–DII interface (spanning residues 251–259). Remarkably, these interactions appeared to be disrupted exclusively at pH 6, whereas they remained partially preserved at pH 5. This observation corroborates our RMSD and dissociation data, suggesting that pH 6 may represent an important destabilizing condition for DENV-3 dimer interface, likely due to the unique histidine configuration discussed previously.

This serotype-specific divergence is further amplified when analyzing specific polar interactions (Figure 7C). For DENV-2, the transition from pH 7 to an acidic environment triggers a widespread occupancy reduction of hydrogen bonds and salt bridges across the dimer interface. Key stabilizing pairs, such as Asp98-Ser7, Asp154-Lys246, Glu62-Lys123, and Glu257-Ser255, are lost or significantly weakened. Conversely, the DENV-3 dimer displays a remarkably resilient polar network; no interactions are completely lost upon acidification. The only notable change is the transient weakening of the Glu267-Lys239 interactions specifically at pH 6, which recover strength at pH 5.

Collectively, these results map a physicochemical landscape where DENV-2 appears to rely on a broad network of pH-sensitive clamps that release progressively down to pH 5, while DENV-3 stability hinges on a focused set of interactions critically perturbed at pH 6. Targeting these specific interfacial “hotspots” to prevent their acid-induced rupture may inform stabilization strategies for developing antibodies and small-molecules against DENV-2 and DENV-3. However, the extent to which similar strategies apply to DENV-1 and DENV-4 will require further investigation.

## 4 Conclusions

In this study, we utilized all-atom and coarse-grained molecular dynamics simulations to investigate how pH and temperature may modulate the conformational dynamics of the DENV-2 and DENV-3 E glycoprotein ectodomains. Our simulations suggest that raising the temperature to 37 °C may expand the conformational space and modulate dimer interface stability through changes in interfacial interactions. However, these observations are based on limited sampling (1 μ s per condition) and require validation through additional simulations and experimental studies. We observed an apparent asymmetric dissociation mechanism, termed the “compensatory embrace”, in 3 out of 18 all-atom trajectories and in coarse-grained models, where the recession of domains in one monomer was counterbalanced by the advance of its partner. However, its biological relevance remains to be determined.

Furthermore, our contact mapping suggests distinct molecular signatures of vulnerability for each serotype. While DENV-2 ecpE dimer stability depended on a broad network of interactions, DENV-3 ecpE dimer integrity was maintained by a more restricted set of contacts, primarily the GLU267–LYS239 interaction. This specific pair, functioning as both a salt bridge and a hydrogen bond, exhibited higher relative stability compared to other interfacial contacts, acting as a persistent structural anchor throughout our simulations. To complement these atomistic insights and test the proposed “compensatory embrace” mechanism, we performed coarse-grained simulations. These simulations consistently reproduced our observations and captured the complete dissociation (in 2 out of 5 replicas) of the dimer in specific systems. Overall, we provided a detailed map of the interactions underlying dimer integrity, establishing a hypothesis-generating framework for the development of antivirals and neutralizing antibodies targeting these serotypes.

## Supplementary Material

















## Data Availability

All simulation input files (including system topologies), output trajectories, and scripts used for the analyses reported in this paper (including procedures for removing periodic boundary condition artifacts) are freely available at Zenodo (10.5281/zenodo.19738169). To reduce data volume, water molecules were removed from the output molecular dynamics trajectories, and the trajectories were subsampled from the original trajectories by saving every 20th frame for all-atom models and every fourth frame for coarse-grained models.

## Notes

Videos S1–S3 show all-atom molecular dynamics trajectories illustrating the “compensatory embrace” mechanism in DENV-2 and DENV-3 under different pH and temperature conditions (MP4)
Videos S2 (MP4)
Videos S3 (MP4)
Videos S4–S6 show trajectories from coarse-grained simulations that illustrate the “compensatory embrace” mechanism in DENV-2 (T37pH5) and DENV-3 (T28pH5 and T37pH7) (MP4)
Videos S5 (MP4)
Videos S6 (MP4)
Video S7 shows the dissociation observed in replicates 01 and 05 in D3T28pH5 and replicates 02 and 05 in D3T37pH7 (MP4)
Figures S1–S7 (PDF)
