# Supplementary material for: Synergistic Effects of pH and Temperature on Dengue Virus Envelope Dimers: Insights from Microsecond Molecular Dynamics Simulations
Source: J Chem Inf Model. 2026 Sep 4;66(20):13467–81. doi: 10.1021/acs.jcim.6c01334 (PMC13715524; doi:10.1021/acs.jcim.6c01334)
Supplement: Supplementary file 1 [file ci6c01334_si_008.pdf]

SUPPORTING INFORMATION

Synergistic Effects of pH and Temperature on Dengue Virus Envelope Dimers: Insights from Microsecond Molecular Dynamics Simulations

Georcki Ropón-Palacios[1], Luís G. F. Crespi[1], Ingrid B. S. Martins[1], Walter Rocchia[2], and Alexandre S. de Araujo*[1]

[1]Department of Physics, São Paulo State University (UNESP), Institute of Biosciences, Humanities and Exact Sciences, São José do Rio Preto, SP, 15054-000, Brazil

[2]CONCEPT Lab, Istituto Italiano di Tecnologia (IIT), Via Enrico Melen, 83 – 16152 Genova, Italy

*Corresponding author:

[1]E-mail: alexandre.suman@unesp.br , Phone: +55 (17) 3221-2566

SUPPORTING INFORMATION

The Supporting Information is available free of charge at the ACS Publications website.

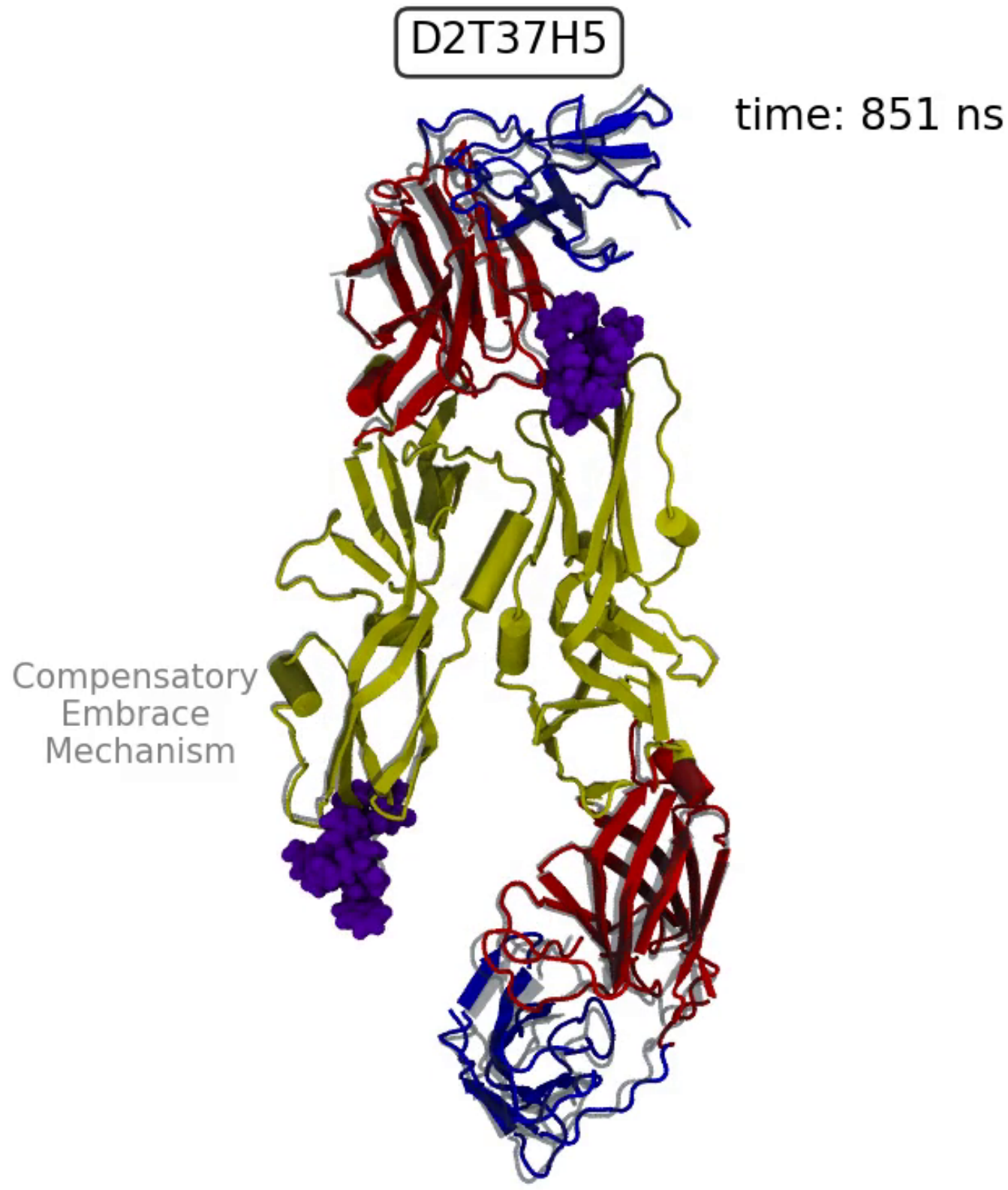

Figure S1. All-atom molecular dynamics trajectory illustrating the "compensatory embrace" mechanism in the DENV-2 envelope dimer under conditions of 37 °C and pH 5 (D2T37pH5).

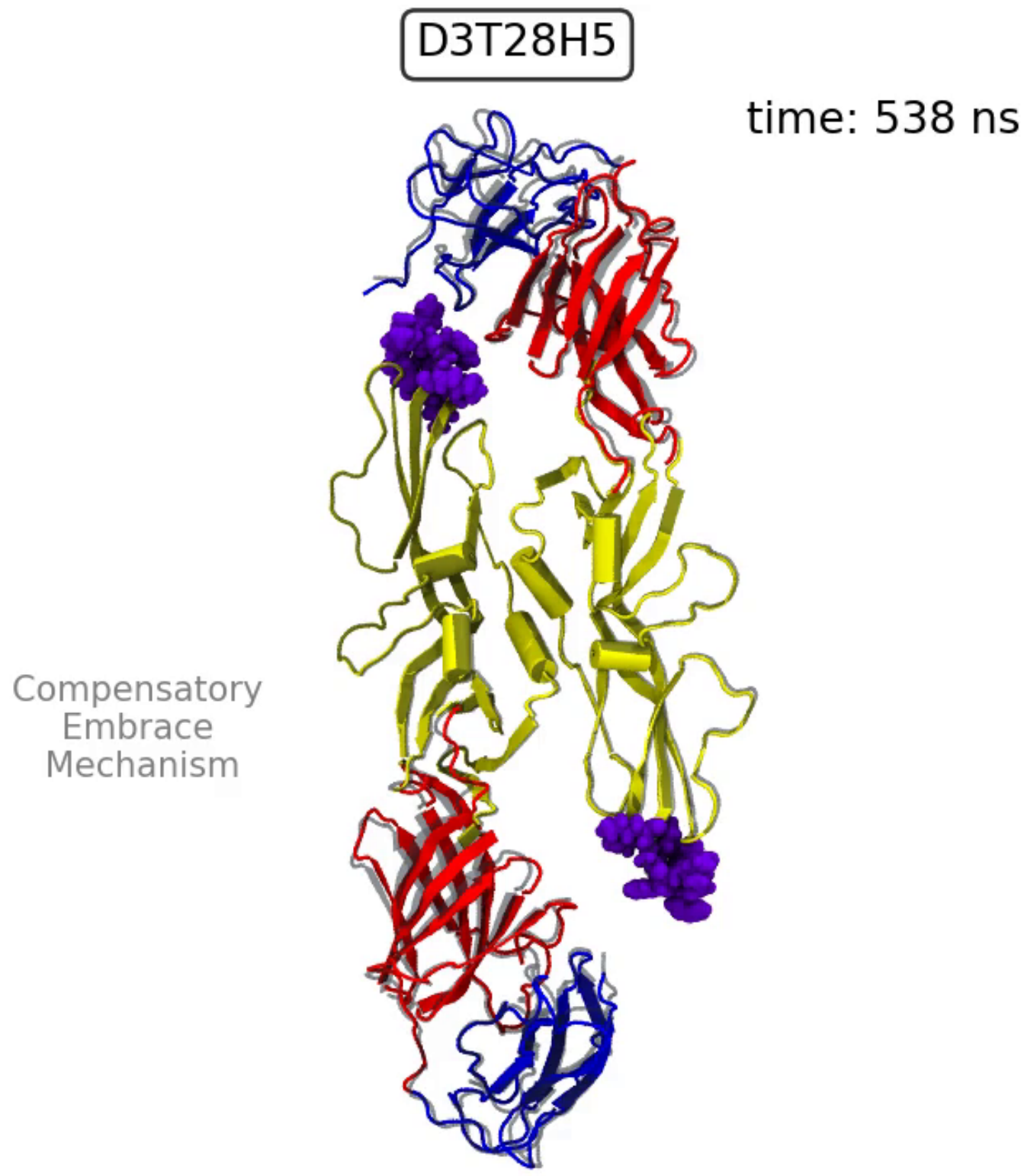

Figure S2. All-atom molecular dynamics trajectory illustrating the “compensatory embrace” mechanism in the DENV-3 envelope dimer under conditions of 28 °C and pH 5 (D3T28pH5).

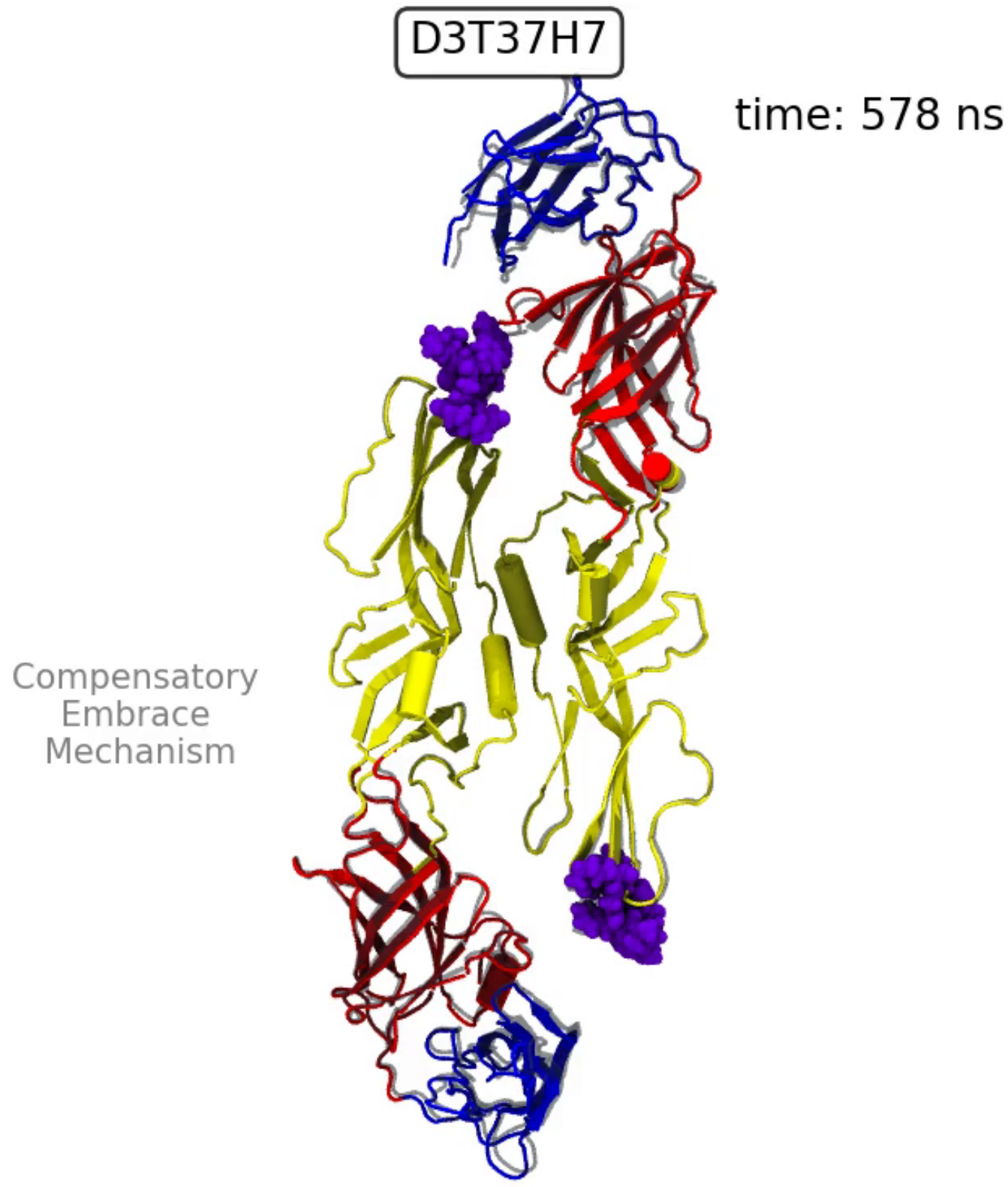

Figure S3. All-atom molecular dynamics trajectory illustrating the "compensatory embrace" mechanism in the DENV-3 envelope dimer under conditions of 37 °C and pH 7 (D3T37pH7).

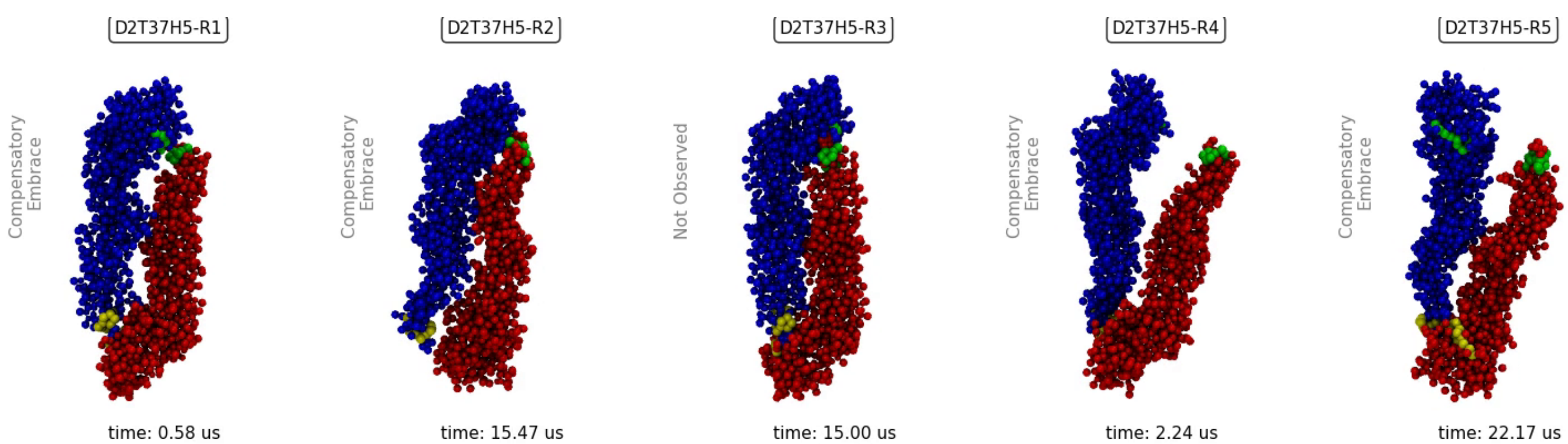

Figure S4. Trajectory from coarse-grained simulations illustrating the "compensatory embrace" mechanism in the DENV-2 envelope dimer at 37 °C and pH 5 (T37pH5).

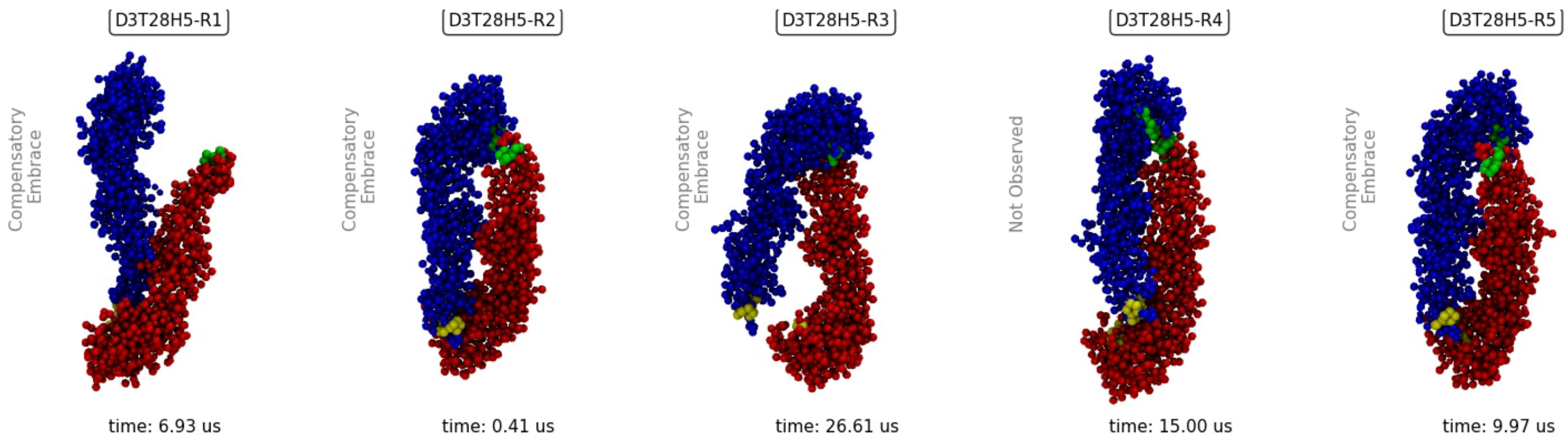

Figure S5. Trajectory from coarse-grained simulations illustrating the "compensatory embrace" mechanism in the DENV-3 envelope dimer at 28 °C and pH 5 (T28pH5).

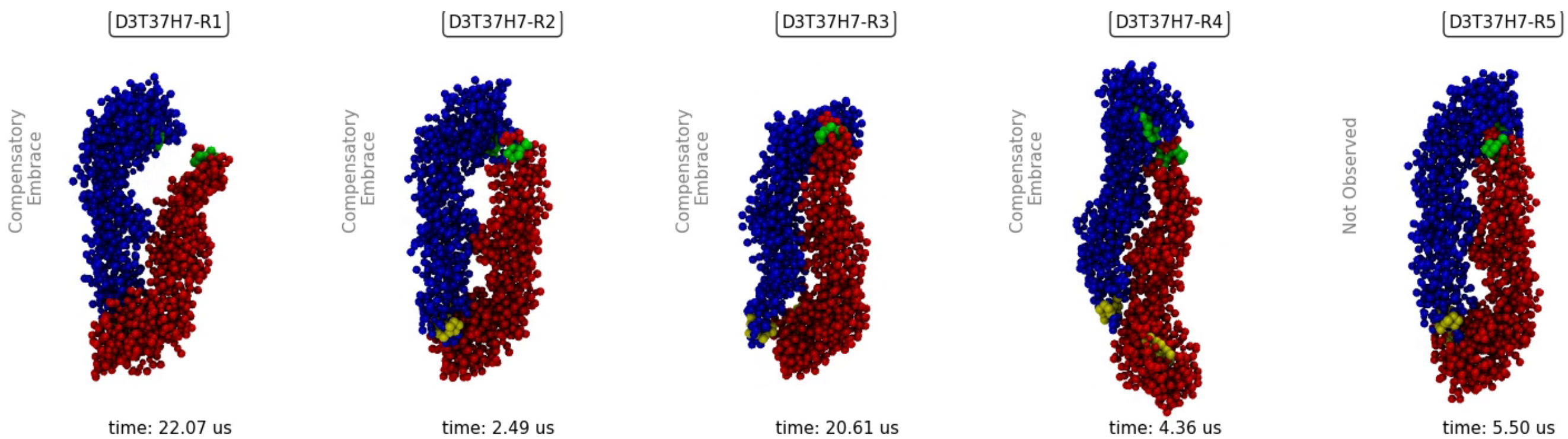

Figure S6. Trajectory from coarse-grained simulations illustrating the "compensatory embrace" mechanism in the DENV-3 envelope dimer at 37 °C and pH 7 (T37pH7).

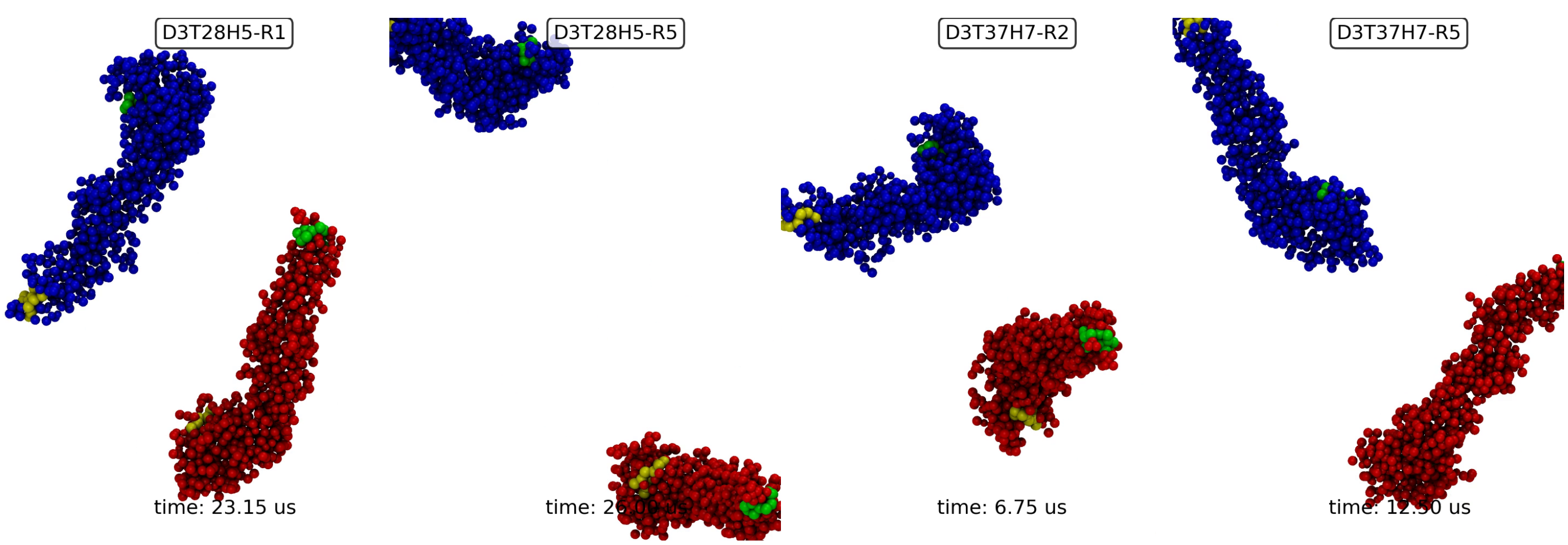

Figure S7. Dissociation events observed during coarse-grained simulations in replicates 01 and 05 for DENV-3 at 28 °C and pH 5 (D3T28pH5), and in replicates 02 and 05 for DENV-3 at 37 °C and pH 7 (D3T37pH7).
